# Clopidogrel Pharmacokinetics in Malaysian Population Groups: The Impact of Inter-Ethnic Variability

**DOI:** 10.3390/ph11030074

**Published:** 2018-07-26

**Authors:** Zaril H. Zakaria, Alan Y. Y. Fong, Raj K. S. Badhan

**Affiliations:** 1 Ministry of Health Malaysia, Block E1, E3, E6, E7 & E10, Parcel E, Federal Government Administration Centre, Putrajaya 62590, Malaysia; zakariz1@aston.ac.uk (Z.H.Z.); alanfong@crc.gov.my (A.Y.Y.F.); 2 Applied Health Research Group, School of Life and Health Sciences, Aston University, Birmingham B4 7ET, UK; 3 Sarawak Heart Centre, Kota Samarahan 94300, Malaysia; 4 Clinical Research Centre, Sarawak General Hospital, Kuching 93586, Malaysia; 5 Aston Pharmacy School, Aston University, Birmingham B4 7ET, UK

**Keywords:** PBPK, pharmacokinetics, clopidogrel, CVD, Malaysian

## Abstract

Malaysia is a multi-ethnic society whereby the impact of pharmacogenetic differences between ethnic groups may contribute significantly to variability in clinical therapy. One of the leading causes of mortality in Malaysia is cardiovascular disease (CVD), which accounts for up to 26% of all hospital deaths annually. Clopidogrel is used as an adjunct treatment in the secondary prevention of cardiovascular events. CYP2C19 plays an integral part in the metabolism of clopidogrel to the active metabolite clopi-H4. However, CYP2C19 genetic polymorphism, prominent in Malaysians, could influence target clopi-H4 plasma concentrations for clinical efficacy. This study addresses how inter-ethnicity variability within the Malaysian population impacts the attainment of clopi-H4 target plasma concentration under different CYP2C19 polymorphisms through pharmacokinetic (PK) modelling. We illustrated a statistically significant difference (*P* < 0.001) in the clopi-H4 C_max_ between the extensive metabolisers (EM) and poor metabolisers (PM) phenotypes with either Malay or Malaysian Chinese population groups. Furthermore, the number of PM individuals with peak clopi-H4 concentrations below the minimum therapeutic level was partially recovered using a high-dose strategy (600 mg loading dose followed by a 150 mg maintenance dose), which resulted in an approximate 50% increase in subjects attaining the minimum clopi-H4 plasma concentration for a therapeutic effect.

## 1. Introduction

Malaysia is a multi-ethnic society with a population of over 32 million that is comprised of three predominant ethic groups, namely Malays (50.1%), Chinese (20.8%) and Indians (6.2%) [1]. In a recent report by the Malaysian National Centre for Adverse Drug Reaction, 13,789 adverse drug reactions were reported during the period from 2015 to 2016 [2]. Given the mixed ethnicity of Malaysia, the impact of pharmacogenetic differences amongst ethnic groups may contribute significantly to the prevalence of toxicity and ineffective clinical therapy [3,4,5].

One of the leading causes of mortality in Malaysia is cardiovascular disease (CVD), which accounts for 22.6% and 26.4% of all hospital deaths annually in Ministry of Health Malaysia hospitals and Malaysian private hospitals, respectively [6]. Among those deaths, ischaemic heart disease accounts for the majority of all reported cardiovascular mortality, followed by acute myocardial infarction. Furthermore, mortality rates have increased steadily since 1990 despite improvements in health services [6].

**Figure 1:**
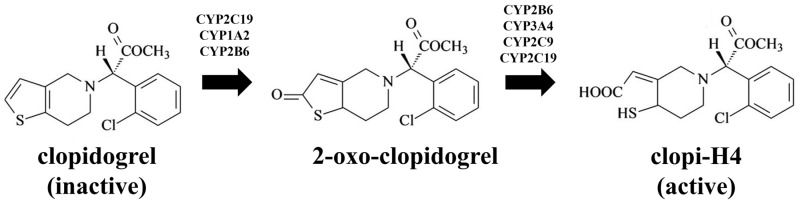
Biotransformation of clopidogrel to the active (H4) metabolite.

Clopidogrel is a second generation thienopyridine antiplatelet drug and a prodrug that is metabolised through two pathways: initially by CYP2B6, CYP1A2 and CYP2C19 leading to the inactive carboxylic acid derivative (2-oxo-clopidogrel), and subsequently by CYP2C19, CYP2C9, CYP2B6 and CYP3A4, leading to its active metabolite (clopi-H4) [7] (Figure 1). The active metabolite confers clopidogrel its therapeutic response by inhibition of adenosine diphosphate-induced aggregation, which in turn activates the irreversible binding of the platelet P2Y12 receptor [8]. The contribution of CYP2C19 towards the formation of clopi-H4 has been further confirmed by several studies [9,10,11] and contributes 45% of the first step and 20% of the second step of total hepatic biotransformation [12,13]. Since CYP2C19 plays an integral part in the metabolism of clopidogrel, any disruption or modification in CYP2C19 expression could potentially affect the pharmacokinetic profiles of clopi-H4, hence leading to effects on its therapeutic response [14].

Clinically, approximately one-fourth of individuals who are treated with clopidogrel exhibit a sub-therapeutic response [15], with the loss-of-function genotype reducing platelet inhibition by clopidogrel [16,17] as a result of reduced clopi-H4 levels [12,18].

More than 50 genetic variants have been identified for CYP2C19 [19]. The wild-type CYP2C19*1 allele is related to functional CYP2C19 metabolism, with CYP2C19*2 and *3 being associated with a lost-of-function (LOF) [20]. Gain-of-function (GOF) variants have also been identified and are primarily related to the CYP2C19*17 variant, which results in higher catalytic activity of CYP2C19 [21]. Thus, individuals presenting with the homozygote and heterozygote allelic variants *2/*2, *3/*3, or *2/*3 are considered to be representative of poor metaboliser (PM) phenotypes; those with variants *1/*2 or *1/*3 (and possibly *2/*17 or *3/*17) are considered intermediate metaboliser (IM) phenotypes; those with *1/*1 are considered wild-type or extensive metaboliser (EM) phenotypes and those with *17/*17 or *1/*17 are considered ultra-rapid metabolisers (UM) [22]. Significant inter-ethnic differences exist in the prevalence of these allelic frequencies, with the CYP2C19*2 [23] and CYP2C19*3 [24] alleles in the broader Asian populations being significantly higher compared to other racial groups [3,25]. This would suggest that Asian population groups would be more likely to be resistant to clopidogrel therapy. In European population groups, EM phenotypes predominate with approximately 30% EM and 2% presenting as PM [26,27,28]. Within the Malaysian population group, Chinese and Malays have broadly similar prevalence of the *1/*1 genotype, 31.6% and 34.5%, respectively [26,27,28,29]. Furthermore, *1/*17 genotypes were broadly similar (3.5% and 3.5% for Chinese and Malays respectively). However, some differences were noted in the prevalence of genotypes, for example, *1/*2 was greater for Chinese (43.9%) compared to Malay (31%) and *1/*3 was higher for Malay (17.2%) compared to Chinese (3.5%) [29].

LOF genotypes can often result in reduced active metabolite plasma concentrations. For example, in a clinical study by Brandt et al. [18], the maximum clopi-H4 plasma concentration (C_max_) for wild-type CYP2C19 subjects (*n* = 56) was 58.4 ± 9.2 ng/mL, compared to CYP2C19*2 carriers, for whom the mean C_max_ was reported to be 35.3 ± 4.3 ng/mL, a 40% decrease in C_max_. Furthermore, pharmacogenetic studies have utilised dose optimisation to counter this reduced clopi-H4 C_max_, whereby a loading dose of 600 mg followed by a maintenance dose of 150 mg could partially restore clopi-H4 to levels observed with a lower loading dose of 300 mg and the standard 75 mg maintenance dose [9].

The advent of personalised medicine has allowed the clinicians to better respond to the impact of genetic variability on clopidogrel therapy. However, such genotyping techniques have met with some contrasting views in relation to their clinic usefulness [30,31]. The impact of anthropometric difference within a diverse patient population group can further confound the understanding of the impact of CYP2C19 genetic variability within mixed populations, and these factored together may significantly alter the pharmacokinetics of drugs. Examples of these factors may include differences within patient demographics (body weight, age, glomerular filtration rate (GFR)), blood biochemistry (plasma proteins, haematocrit) and drug metabolism enzyme abundances (CYP abundance and polymorphism).

Precision medicine allows an individual’s unique physiological characteristics to be incorporated into treatment options, whereby treatments are tailored to individual patients based on their individual genetic, biomarker, phenotypic, and psychosocial characteristics [32,33]. To assist in the process of integrating such a diverse range of anthropometric and genetic factors into clinical decision-making, the application of pharmacokinetic modelling and simulation has emerged as techniques to better individualise drug therapy. In particular, the field of population-based physiologically based pharmacokinetic (PBPK) modelling has rapidly gained traction by drug regulatory authorities and the wider pharmaceutical industry as a viable means to ‘simulate’ clinical trials and the pharmacokinetics of drug compounds within virtual population groups representative of individual population groups [34,35,36,37,38,39]. Furthermore, the application of PBPK modelling can allow for the use of population-specific anthropometric variability within virtual subjects, and this was recently demonstrated by our group when considering the optimisation of anti-malarial therapy in sub-Saharan African population groups using PBPK-based virtual clinical trials, where the population groups incorporated anthropometric and biochemical alterations from standard ‘healthy volunteer’ clinical trials subjects [40,41].

To our knowledge, we present here the first application of PBPK modelling to develop a Malaysian population group for the specific purpose of understating the impact of genotype drug therapy within this mixed-ethnicity population. The study directly addresses this inter-ethnicity variability and provides a research tool that brings together the complexity (at a cellular level) of systems-biology with the ease-of-use applicability of pharmacokinetic modelling to provide a robust predictive platform that can easily be adapted and developed as required within the Malaysian population. The objectives of the present study were two-fold: (i) to predict clopidogrel pharmacokinetics in the Malay and Malaysian Chinese adult population groups and (ii) to address the impact of the *1/*1, *2/*2, *1/*2 and *1/*17 CYP2C19 genotype on clopidogrel pharmacokinetic.

## 2. Results

### 2.1. Step 1: Malaysian Population Group Development

#### 2.1.1. The National Cardiovascular Disease Database

The three largest population groups were selected for analysis and identified as Malay, Chinese and Indian, with Malay comprising the largest ethnic group contained within the National Cardiovascular Disease (NCVD) database (Table 1). The mean age, weight and BMI were significantly different between Malay and Malaysian Chinese (*p* < 0.0001) and Malaysian Chinese and Malaysian Indian (*p* < 0.0001), whereas mean height was relatively consistent across all population groups (1.63 m) and is not statistically significantly different (Table 1).

#### 2.1.2. Development of Age–Weight Relationships for Malaysian Populations

Malaysian population groups were subsequently developed for the Malay and Malaysian Chinese groups. Polynomial mathematical relationship for gender-specific age–weight relationships for the Malay population group are described in Equations (1) and (2) for 20–65 year olds:

Malay male body weight = −786.757075 + (−105.598305 × age) + 9.79604022 × age^1.5^ + (−0.33871491 × age^2^) + 498.1612119 × age^0.5^, (1)

Malay female body weight = −3348.57622 + 2424.271248 × age^0.5^ + (−676.182360 × age) + 92.98417478 × age^1.5^ +( −6.31405170 × age^2^) + 0.169288237 × age^2.5^. (2)

**Figure 2:**
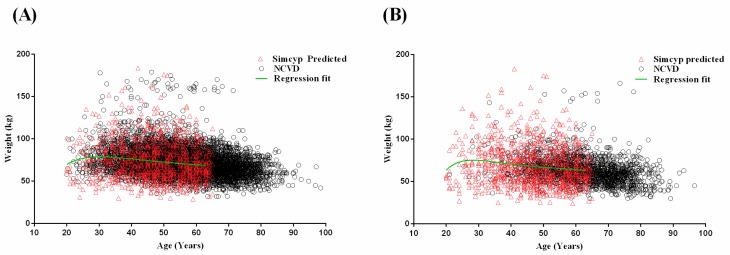
Visual predictive checks for the comparison between predicted and observed (NCVD) age–weight relationship for Malay male (*n* = 18,601) (**A**) and female (*n* = 4513) (**B**) populations. Red outlined triangles represent the Simcyp predicted population age–weight relationships. Black outlined circles represent the observed population age–weight relationships from the NCVD database. Green lines represent the fitted trend-line from the polynomial mathematical relationship.

Visual predictive checks confirmed that model predicted age–weight relationships retained the same distribution across age ranges when compared to the NCVD for Malay males (Figure 2A) and females (Figure 2B).

The polynomial mathematical relationship for gender-specific age–weight relationships for the Malaysian Chinese population group is described in Equations (3) and (4) for 20–65 year olds:

Malaysian Chinese male body weight = (75.50929026 + (−3.86906581 × age) + 0.034908233 × age^2^ + 0.001047109 × age^3^)/(1 + (−0.04452164 × age) + 0.0000141817 × age^2^ + 0.0000206378 × age^3^), (3)

Malaysian Chinese female body weight = 67.51927661 + (−0.00194867 × age^2^) + 0.0000000434656 × age^4^. (4)

**Figure 3:**
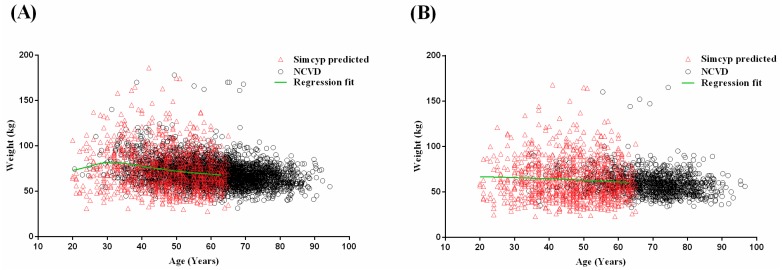
Visual predictive checks on the comparison between predicted and observed (NCVD) age–weight relationship for Chinese male (*n* = 7445) (**A**) and female (*n* = 2484) (**B**) population. Red outlined triangles represent the Simcyp predicted population age–weight relationships. Black outlined circles represent the observed population age–weight relationships from the NCVD database. Green lines represent the fitted trend-line from the polynomial mathematical relationship.

Visual predictive checks were performed to assess the graphical qualification between the polynomial mathematical relationship for age and weight relationships of the software and the observed Malaysian Chinese male and female populations (Figure 3), and confirmed that model predicted age–weight relationships retained the same distribution across age ranges when compared to the NCVD (Figure 3).

### 2.2. Step 2: Adult Simulations: Validation with Repaglinide, Tramadol and Rosuvastatin

#### 2.2.1. Repaglinide

**Figure 4:**
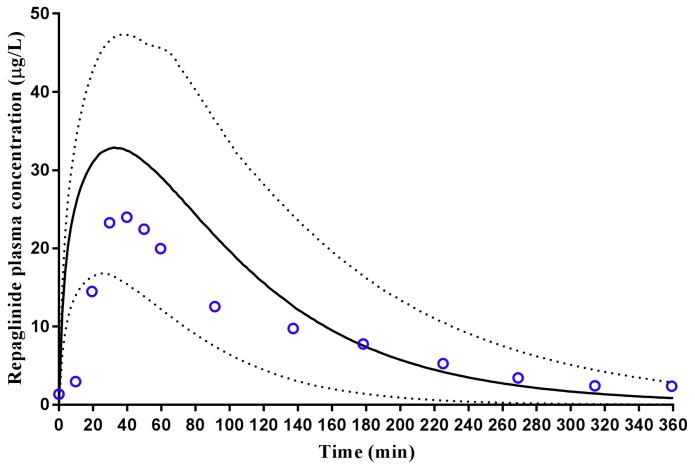
Simulated plasma concentration–time profile of repaglinide in healthy adults. A 2 mg oral dose of repaglinide was administered once daily to healthy adult volunteers (*n* = 24). Solid lines represent mean population prediction with dotted lines representing 5th and 95th percentile ranges. Open circles represent data for the observed study [42].

The repaglinide compound file within the Simcyp library was used in conjunction with the Simcyp ‘Healthy Volunteer’ population group to predict the plasma concentration–time profile for a single 2 mg oral dose of repaglinide in healthy Caucasian subjects. The resultant predictions were within the range of observed reported values (Figure 4) with model predicted t_max_ and C_max_ within two-fold of that reported and AUC within 2.15-fold of the reported (Table 2).

**Figure 5:**
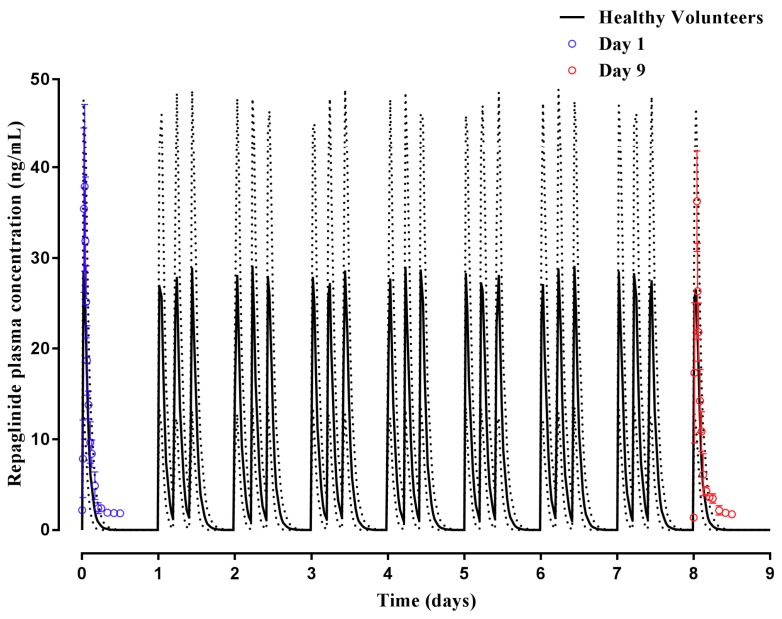
Simulated plasma concentration–time profile of repaglinide following single and multiple doses in healthy adults. An oral dose of 2 mg was administered once daily on day 1, and thereafter daily for nine days to healthy adult volunteers (*n* = 12). Solid lines represent mean predictions with dotted lines representing the 5th and 95th percentile ranges. Open circles represent data for the observed study at day 1 (single dose) and day 9 (multiple-dosing) [43].

Subsequently, to further validate model simulations, the ability to predict repaglinide plasma concentrations following single and multiple dosing was assessed using a healthy volunteer population group. Predicted plasma concentrations following a single dose (day 1) and multiple doses (day 9) were within the range reported [43] (Figure 5), with model predicted t_max_, C_max_ and AUC within two-fold of those reported [43] (Table 2). However, the terminal elimination phase was poorly predicted.

**Figure 6:**
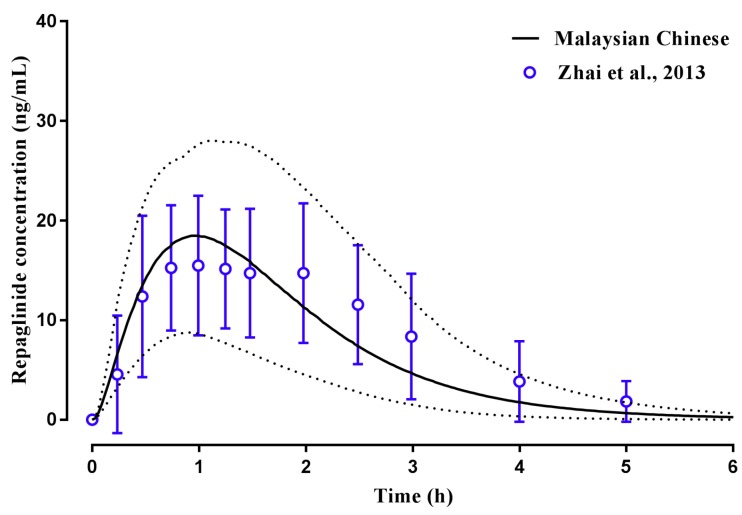
Simulated plasma concentration–time profile of repaglinide in healthy Malaysian Chinese adults. A 2 mg oral dose of repaglinide was administered once daily to adult healthy Chinese volunteers (*n* = 22) using the Malaysian Chinese population group. Solid lines represent mean predictions with dotted lines representing the 5th and 95th percentile ranges. Open circles represent data for the observed study [44].

The model was then extended to assess its application within Malay and Malaysian Chinese population groups. For the Malaysian Chinese population, we utilised the customised Malaysian Chinese population group in the model to predict repaglinide plasma concentrations following a single 2 mg oral dose (Figure 6). The predicted repaglinide plasma concentration was within the range reported with model predicted t_max_, C_max_ and AUC within two-fold of those reported [43] (Table 2).

**Figure 7:**
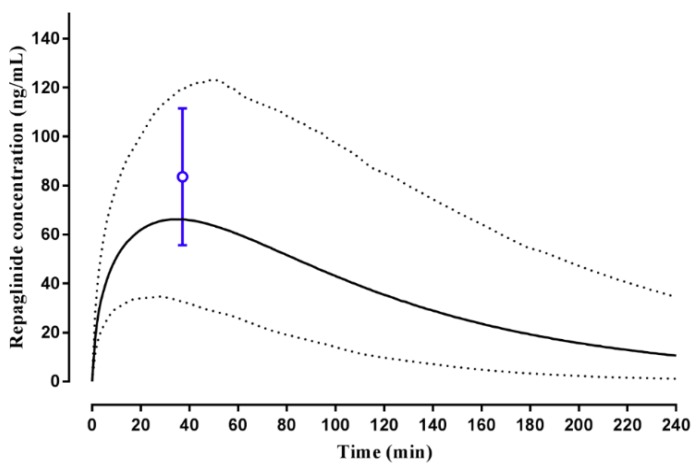
Simulated plasma concentration–time profile of repaglinide in healthy Malay adults. A 4 mg oral dose of repaglinide was administered once daily to adult healthy Malay volunteers (*n* = 121) using the custom Malay Simcyp population group. Solid lines represent mean predictions with dotted lines representing the 5th and 95th percentile ranges. Open circles represent data for the observed study [45].

For the Malay population group, model predicted C_max_ and t_max_ were within the range reported (Figure 7) with clearance (CL) predictions within two-fold of that reported and AUC 2.25-fold of that reported [45] (Table 2).

#### 2.2.2. Tramadol

**Figure 8:**
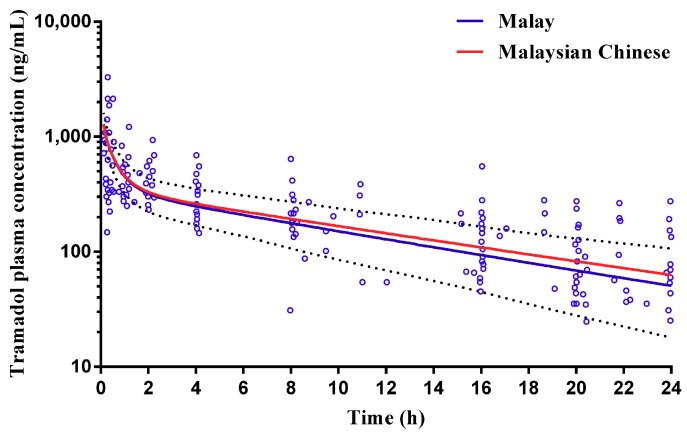
Simulated plasma concentration–time profile of tramadol intravenous bolus dosing in Malay and Malaysian Chinse subjects. An IV-bolus dose of 100 mg tramadol was administered to adult healthy mixed Malaysian volunteers (*n* = 100) using the custom Malaysian (Malay and Malaysian Chinese) Simcyp population group. Solid lines represent mean predictions with dotted lines represent the 5th and 95th percentile ranges. Open circles represent data for the observed study [46].

Tramadol pharmacokinetics have been reported in mixed Malaysian subjects following an IV bolus dose [46]. Furthermore, T’jollyn et al. [47] have developed and validated a tramadol compound within Simcyp, and these studies were used as the basis for predicting tramadol pharmacokinetics in the Malay and Malaysian Chinese populations. Following a 100 mg IV-bolus dose of tramadol, simulated plasma concentrations for Malays and Malaysian Chinese were within the range reported by Gan et al. [46] (Figure 8) with model predicted CL and AUC within two-fold of that reported (Table 2).

#### 2.2.3. Rosuvastatin

**Figure 9:**
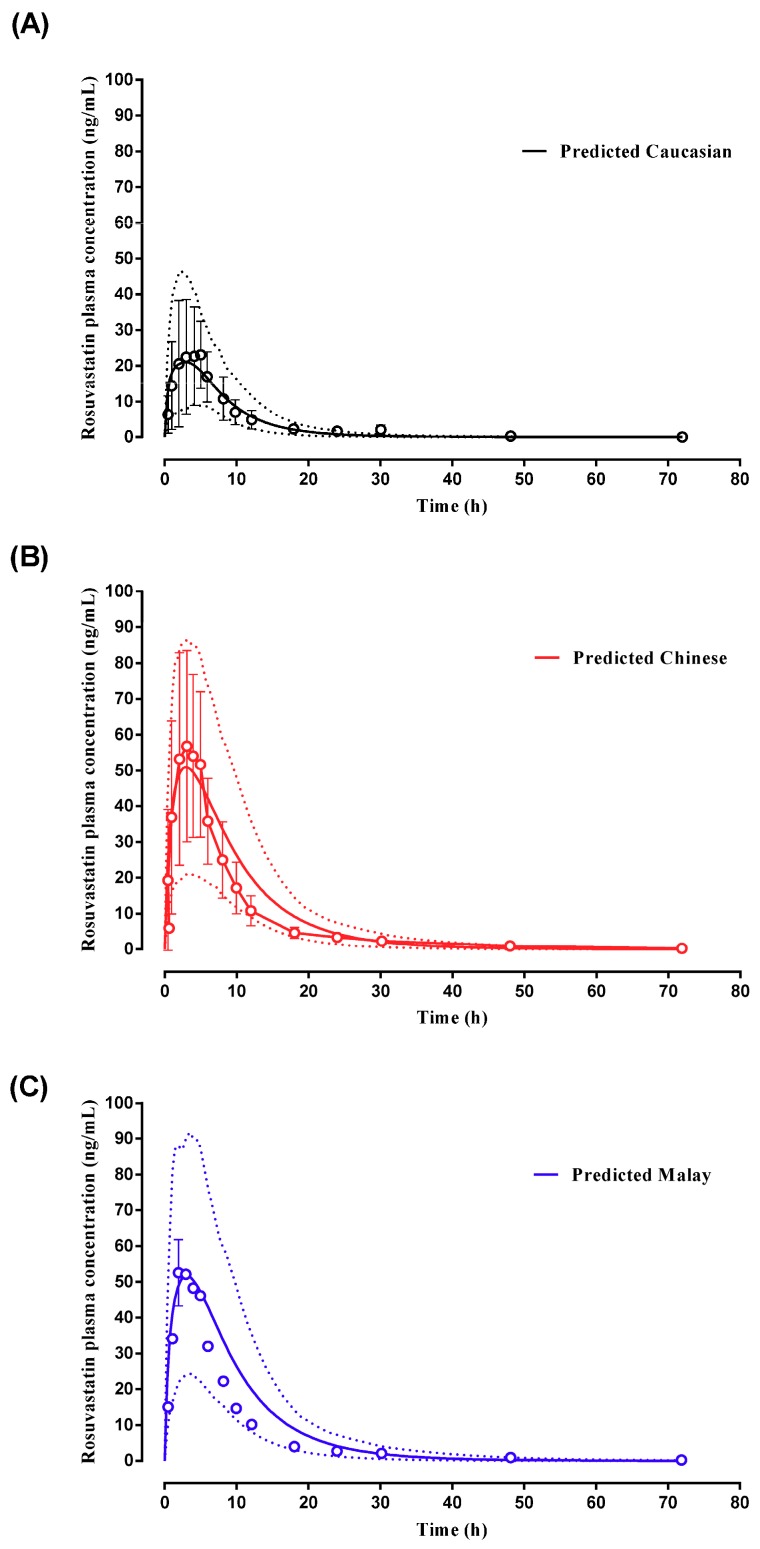
Simulated plasma concentration–time profile of rosuvastatin in (**A**) Caucasian, (**B**) Chinese and (**C**) Malay subjects. A single 40 mg oral dose was administered to adult population groups (*n* = 36 for each population group, matching the reported study recruitment for each population group) using the Simcyp Heathy Volunteer (Caucasian), Simcyp Healthy Chinese (Chinese) and custom Malay population groups. Solid lines represent mean predictions with dotted lines representing the 5th and 95th percentile ranges. Open circles represent mean data for the observed study with error bars indicating standard deviation where reported by Lee et al. [48] (and largely missing in results presented by Lee et al. for Malay subjects).

Rosuvastatin pharmacokinetics have been reported in Caucasian and Asian (Chinese and Malay) subjects following a 40 mg oral dose [48]. Using the rosuvastatin compound within the Simcyp library, simulated plasma concentration–time profiles for all subjects were within the range reported by Lee et al. [48] (Figure 9), with model predicted C_max_, t_max_ and AUC within two-fold of that reported (Table 2).

### 2.3. Step 3: Prediction of the Impact of CYP2C19 Polymorphisms on Clopidogrel Pharmacokinetics in Malaysians

To assess the impact of CYP2C19 SNPs on target clopi-H4 plasma concentrations (0.81–13.45 ng/mL) [49] simulations were conducted in Malay and Malaysian Chinese populations to assess the number of subjects within this therapeutic window. For these simulations, the lowest range of 0.81 ng/mL was used as a cut-off value to depict patients who were unresponsive to clopidogrel treatment. A loading dose of 300 mg was administered on day 1 and a maintenance dose of 75 mg once daily commenced from day 2 to day 5 and was administered to 400 subjects, each possessing either *1/*1, *2/*2, *1/*2 or *1/*17 genotypes (40 × 10 trials, 100 subjects per genotypes). This was conducted for both the Malay and the Malaysian Chinese population groups.

**Figure 10:**
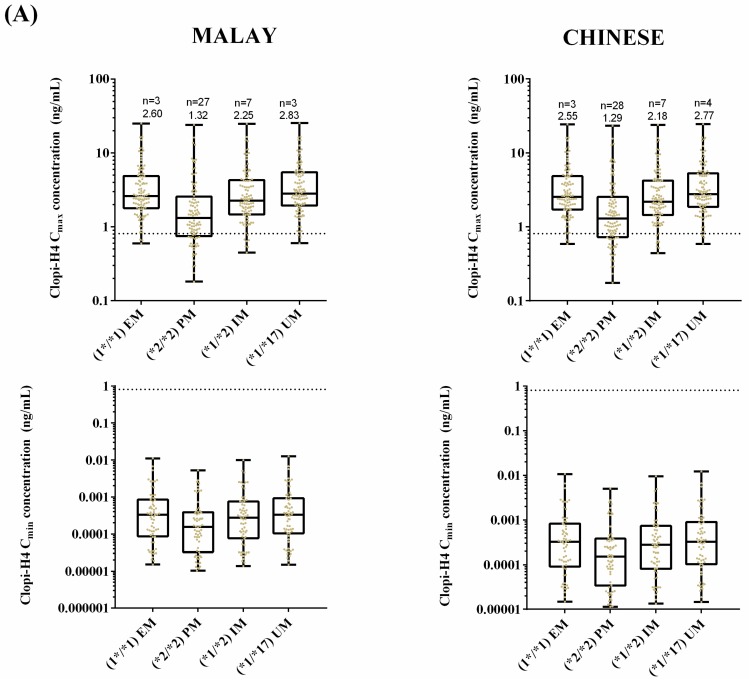

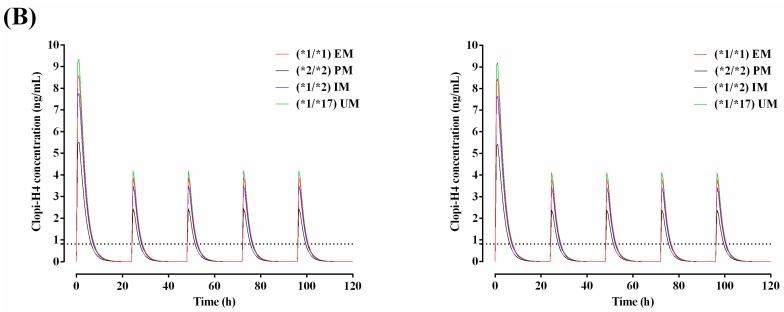
(**A**) impact of clopidogrel standard dose regimens on final dose clopi-H4 plasma C_max_ (upper panels) and C_min_ (lower panels) in Malay (left panels) and Malaysian Chinese (right panels) subjects, demarked for all EM, PM, IM or UM populations. Box and whisker plots represent maximum, 75th percentile, median, 25th percentile and minimum clopi-H4 C_max_; (**B**) simulated mean plasma concentration–time profile of clopi-H4 in Malay (left panel) and Malaysian Chinese (right panel) subjects demarked for all EM, PM, IM or UM populations. All subjects (*n* = 100 for each phenotype) received a loading dose of 300 mg on day 1, followed by daily doses of 75 mg for four days. Solid lines represent mean plasma concentration–time predictions. Dashed horizontal lines represent the lower therapeutic limit for clopi-H4.

Within the Malay population, three subjects of *1/*1 genotype did not reach the target concentration, followed by 27 subjects with the *2/*2 genotype, 7 subjects with the *1/*2 genotype and three subjects with the *1/*17 genotype (Figure 10). In the Malaysian Chinese population, the number of subjects who did not reach the target concentration followed a similar pattern to that of the Malay population, with three subjects for the *1/*1 genotype, 28 subjects for the *2/*2 genotype, seven subjects for the *1/*2 genotype and four subjects for the *1/*17 genotype (Figure 10).

There were no statistically significant differences between Malay and Malaysian Chinese when comparing clopi-H4 C_max_ or C_min_ concentrations in each phenotype (Figure 10). However, as expected, in both Malay and Malaysian Chinese populations, the *2/*2 (PM) phenotype resulted in a statistically significant difference in mean clopi-H4 C_max_ when compared to all other phenotypes (*p* < 0.001).

### 2.4. Step 4: Sensitivity Analysis for CYP2C19 Hepatic Abundances

**Figure 11:**
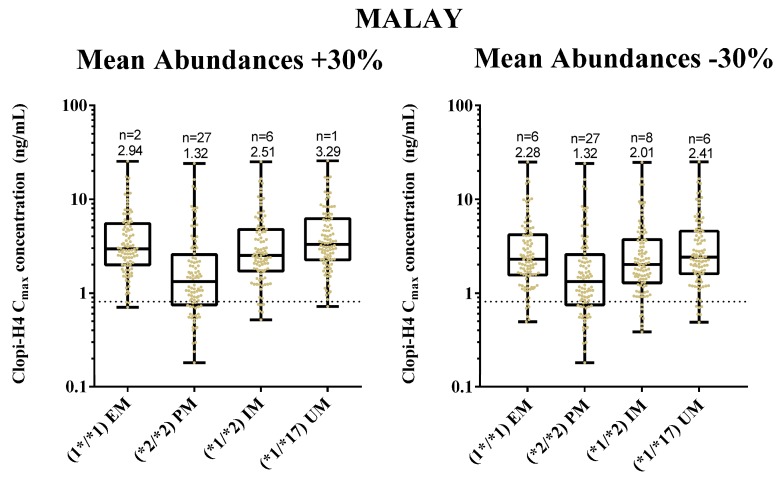

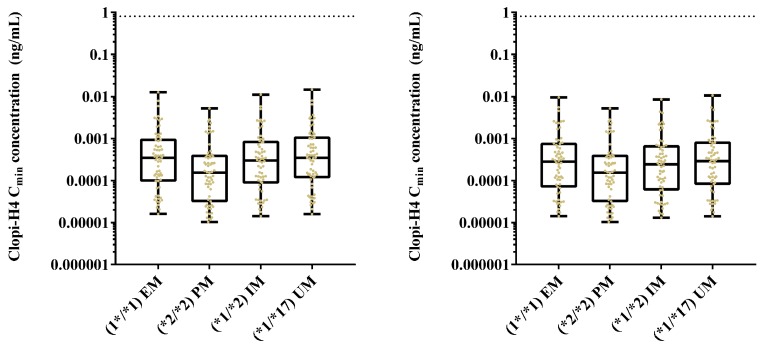
Impact of clopidogrel standard dose regimens on final dose clopi-H4 plasma C_max_ (upper panels) and C_min_ (lower panels) in Malay subjects under scenarios where mean hepatic CYP2C19 abundance is increased (left panels) or decreased (right panels) by 30%. All subjects are demarked for either all EM, PM, IM or UM populations. Box and whisker plots represent maximum, 75th percentile, median, 25th percentile and minimum clopi-H4 C_max_. All subjects (*n* = 100 for each phenotype) received a loading dose of 300 mg on day 1, followed by daily doses of 75 mg for four days. Dashed horizontal lines represent the lower therapeutic limit for clopi-H4.

Considering the lack of literature on reported CYP2C19 hepatic abundance data for Malays, the sensitivity of model predictions to changes in CYP2C19 hepatic abundance (assuming they varied from the default assumption that Chinese and Malay CYP2C19 abundances were similar) was assessed through simulating the number of subjects attaining target clopi-H4 concentrations when the hepatic mean enzyme abundances were increased or decreased by 30%. In the Malay population, a 30% increase in mean abundance values resulted in 2, 27, 6 and 1 subjects failing to reach the target concentration for the *1/*1, *2/*2, *1/*2 and *1/*17 genotypes, respectively (Figure 11). Furthermore, a 30% decrease of mean abundance values resulted in 6, 27, 8 and 6 subjects failing to reach the target concentration for the *1/*1, *2/*2, *1/*2 and *1/*17 genotypes, respectively (Figure 11).

**Figure 12:**
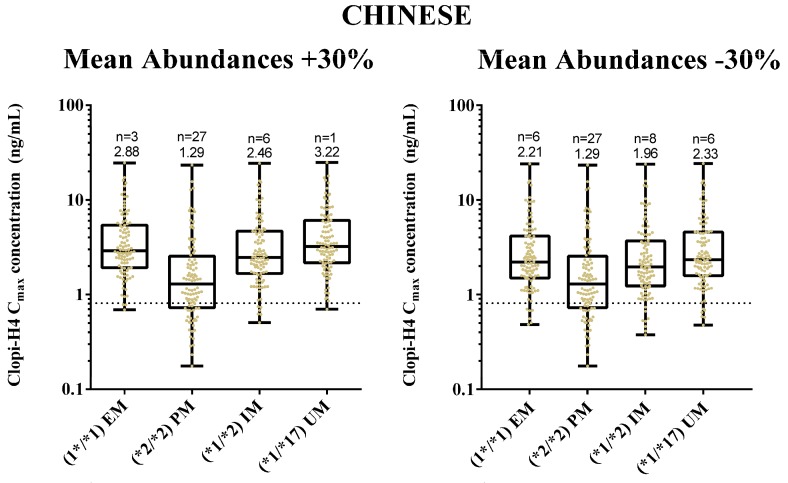

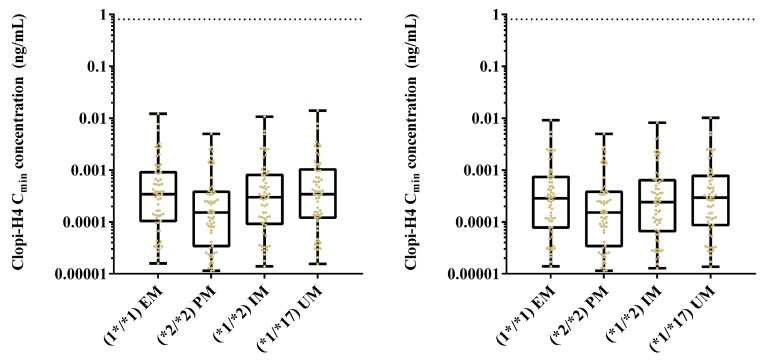
Impact of clopidogrel standard dose regimens on final dose clopi-H4 plasma C_max_ (upper panels) and C_min_ (lower panels) in Chinese subjects under scenarios where mean hepatic CYP2C19 abundance is increased (left panels) or decreased (right panels) by 30%. All subjects are demarked for either all EM, PM, IM or UM populations. Box and whisker plots represent maximum, 75th percentile, median, 25th percentile and minimum clopi-H4 C_max_. All subjects (*n* = 100 for each phenotype) received a loading dose of 300 mg on day 1, followed by daily doses of 75 mg for four days. Dashed horizontal lines represent the lower therapeutic limit for clopi-H4.

For the Malaysian Chinese population, a 30% increase in the mean abundances values resulted in 3, 27, 6 and 1 subjects failing to reach the target concentration for the *1/*1, *2/*2, *1/*2 and *1/*17 genotypes, respectively (Figure 12). Furthermore, a 30% decrease in the mean abundance values resulted in 6, 27, 8 and 6 subjects failing to reach the target concentration for the *1/*1, *2/*2, *1/*2 and *1/*17 genotypes, respectively (Figure 12). There were also no statistically significant inter-ethnic differences between the clopi-H4 peak and trough concentrations for each genotype.

### 2.5. Step 5: Dose Optimization in CYP2C19 Poor Metabolisers

**Figure 13:**
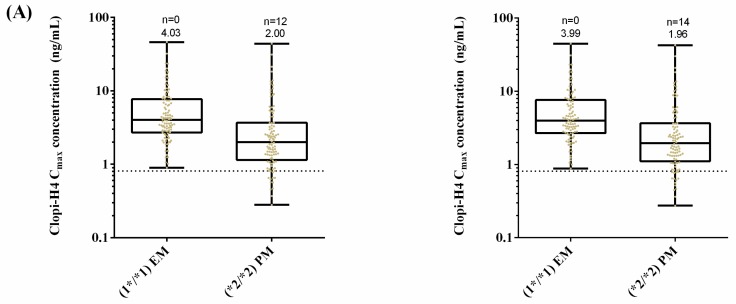

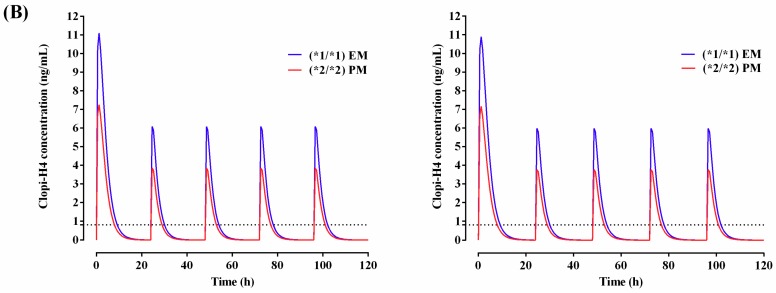
(**A**) impact of clopidogrel high dose regimen on clopi-H4 plasma C_max_ in Malay (left panel) and Malaysian Chinese (right panel) subjects; (**B**) simulated mean plasma concentration–time profile of clopi-H4 in Malay (left panel) and Malaysian Chinese (right panel) subjects. An oral loading dose of 600 mg was administered on day 1, followed by daily doses of 150 mg for four days using the custom Malaysian (Malay and Malaysian Chinese) Simcyp population group (*n* = 100), with dosing to populations of either all PM or all EM phenotypes. Solid plasma concentration lines represent mean predictions. Dashed horizontal lines represent the lower therapeutic limit for clopi-H4. Box and whisker plots represent maximum, 75th percentile, median, 25th percentile and minimum clopi-H4 C_max_.

Given the high number of subjects identified with clopi-H4 concentrations below the target threshold (Figure 10) in the PM phenotype group, the dosing regimen for clopidogrel was increased to a ‘high-dose’ scenario with a 600 mg loading dose followed by a four-day regimen of 150 mg daily (Figure 13). For a standard dose, 27 Malay and 28 Malaysian Chinese subjects had a clopi-H4 plasma concentration below the target minimum therapeutic concentration (Figure 10), which decreased to 12 (Malay) and 14 (Malaysian Chinese) for the high dose regimen (Figure 13A). No statistically significant differences were determined between Malay and Malaysian Chinese clopi-H4 C_max_ (Figure 13B).

## 3. Discussion

Cardiovascular disease (CVD) is a leading global cause of mortality with recent reports highlighting that approximately 85% of CVD cases occur in low- to middle-income countries [50,51]. A primary cause of this increase is related to changes in economic development and lifestyle with reduced incidences of infectious diseases, all of which has led to a marked improvement in the life expectancy of low- to middle-income countries from 61.7 years in 1980 to 71.8 years in 2015 [50]. Furthermore, the higher incidences of non-communicable disease, such as diabetes mellitus, hypertension and dyslipidaemia, have all contributed to an overall increase in the incidence of CVD in low- to middle-income countries.

Despite these risks, the Ministry of Health (MOH) Malaysia has a proactive stance in relation to CVD and has maintained a long-standing database of cardiovascular disease in Malaysia, which is utilised to evaluate risk factors and treatment within Malaysia. The National Cardiovascular Database (NCVD) Registry [6] ensures the ongoing systematic collection, analysis and interpretation of cardiovascular disease data essential, which is essential and core to planning, implementation and evaluation of clinical and public health services within Malaysia. The NCVD was officially launched in 2006 by Dr. Ghani Mohamed Din (Deputy Director General of Health Malaysia) during the 10th Annual Scientific Meeting (ASM) of the National Heart Association of Malaysia (NHAM). To date, the NCVD register consists of 33,043 anonymised and voluntary patient records for patients undergoing acute coronary syndrome and percutaneous coronary intervention, spanning the years 2006–2015. 

Although aspirin remains a primary treatment option for many CVD related disorders due to its cost-effectiveness [52], a key reason for clinicians moving towards a second-line therapy is often hypersensitivity of patients towards aspirin. In Malaysia, clopidogrel is recommended as a second-line therapy for ischemic cardiovascular events and also secondary prevention of ischemic stroke [53,54]. Clopidogrel is an antiplatelet agent, being predominantly metabolised into the active metabolite clopi-H4, primarily mediated by CYP2C19 [9,10,11]. However, CYP2C19 is highly polymorphic [55], with the PM phenotypes (*2/*2) known to be of higher prevalence in Asian populations when compared to Caucasians [56,57]. 

Outside of Southeast Asia, the use of predictive pharmacokinetics modelling to aid in both drug discovery and development along with clinical optimisation of drug therapy has exponentially increased over the past decade and has become a routine aspect of all clinical trials phases to both extrapolate dose to optimal therapy in population groups and to also identify covariates that may contribute to the variability in clinical response to drugs [58,59,60]. However, within Malaysia, the use of pharmacokinetic modelling to conduct such beneficial models’ approaches is lacking.

Recently, we demonstrated the ability of physiologically-based pharmacokinetic modelling to optimise dosing of antimalarial drugs in special population groups from sub-Saharan African nations [40,41,61], where the unique physiological and anthropometric differences of African subjects (when compared to Caucasians) were incorporated into simulations. We have adapted this approach to now develop, for the first time, an appropriate virtual population group of the Malaysian population group for use in mechanistic pharmacokinetic modelling, with a focus on predicting the impact of CYP2C19 SNPs on clopidogrel and the active metabolite, clopi-H4, pharmacokinetic in the Malay and Malaysian Chinese population groups.

We adopted a robust 5-stage modelling approach that incorporated key data from the Malaysian NCVD database to develop virtual population groups, following validation of the modelling approaches using repaglinide, tramadol and rosuvastatin within healthy volunteers (Caucasian), Chinese and Malay population (Steps 1 and 2), followed by the simulation of clopidogrel and its active metabolite, clopi-H4 in the Malay and Malaysian Chinese population groups (Step 3). Next, the impact of CYP2C19 SNPs on the active metabolite, clopi-H4 in the Malay and Malaysian Chinese population groups were assessed with predictions of potential exposure to the clopidogrel therapy (Step 4). Finally, simulations were conducted to predict the potential impact of dosage optimisation in the CYP2C19 PM population (Step 5).

### 3.1. Step 1: Malaysian Population Development

In Step 1, we attempted to develop a representative Malaysian population by extracting relevant data such as gender, weight, age and ethnicity from the NCVD database [6], with which to develop the populations. For the Malay, Chinese and Indian population, the mean age, weight and BMI were significantly different between Malay and Chinese (*p* < 0.0001) and Chinese and Indian (*p* < 0.0001), whereas mean height was relatively consistent across all population group at 1.63 m and not statistically significantly different (Table 1). Since there was no significant difference between the heights of all the three populations, the default age–height relationship for the ‘Chinese Volunteer’ population group within Simcyp was used to represent these populations. Using the customised age–weight relationships for the two populations, the predicted distribution of body-weight with age for both the Malay (Figure 2) and Malaysian Chinese (Figure 3) populations was predicted well and in good agreement with individual subject data extracted from the NCVD data over the range of 20 to 65 years of age. This supports the development of appropriate population groups possessing suitable anthropometric age–weight relationship within the Malaysian population. In addition, this step incorporated appropriate blood biochemistry metrics to describe Malay and Malaysian Chinese population groups, something which is critical for driving unbound drug fraction within plasma and essential as clopidogrel, and its metabolites are extensively protein bound (see Section 4.1.1 Table 3) [62,63].

### 3.2. Step 2: Adult Simulations: Validation with Repaglinide, Tramadol and Rosuvastatin

Having established a Malaysian virtual population group for use in predictive pharmacokinetic modelling, we subsequently assessed the ability of the customized population groups to predict repaglinide and tramadol plasma concentrations and pharmacokinetics in the Malay (Figure 7), Chinese (Figure 6) and Caucasian Healthy Volunteers (Figure 4 and Figure 5) populations. In these simulations, model predictions were successfully predicted to within two-to-three-fold, C_max_, t_max_, CL and AUC was reported (Table 2) [44,45], and an appropriate population distribution was recapitulated. It was noted, however, that in Caucasians the model predicted a poorer terminal elimination phase of the plasma concentration–time profile when multi-dosing (Figure 5). This may, in part, be a result of the original study reporting plasma concentration–time profiles without using a log-linear scale, making precise determination of terminal points difficult. However, it should be noted that the AUC was predicted to within two-fold of that reported [43].

Subsequently, the customised Malay and Malaysian Chinese population group were further validated against a study whereby an IV bolus dose of tramadol was dosed to Malaysian subjects [46]. Model prediction AUC and Clearance were within two-fold of that reported (Table 2) with model predicted plasma concentration–time profiles spanning an appropriate range for the simulated population group when compared to the observed data (Figure 8). Finally, model validation was further confirmed through successful prediction of rosuvastatin plasma concentrations and pharmacokinetics in Caucasian, Chinese and Malays (Figure 9) with predicted C_max_, t_max_, and AUC to within two-fold of that reported [48] (Table 2).

### 3.3. Step 3: Prediction of the Impact of CYP2C19 Polymorphisms on Clopidogrel Pharmacokinetics in Malaysians

Having successfully validated the predicting capability of the customised population groups within pharmacokinetic models for repaglinide and tramadol, the model was expanded to assess its application to predict clopidogrel and its active metabolite, clopi-H4, pharmacokinetics in Malaysian subjects. In an attempt to establish the potential impact of CYP2C19 SNPs on clopi-H4 plasma concentration in Malay and Malaysian Chinese populations, we conducted simulations stratified across EM, PM, IM, and UM phenotypes. A statistically significant difference in the clopi-H4 C_max_ was predicted between the EM and PM groups within the Malay and Malaysian Chinese population (Figure 10), with clopi-H4 C_max_ decreasing by approximately 50% in the PM population groups compared to the EM population group in both Malay and Malaysian Chinese (Figure 10). 

In a study by Simon et al. [9], clopi-H4 plasma concentrations were quantified for each phenotype in European subjects. Following a standard dose (300 mg loading dose followed by 4 days of 75 mg once daily), the last dose mean C_max_ was 13 ± 7.33 ng/mL for EM and 3.93 ± 1.93 ng/mL for PM. In both Malay and Chinese subjects, the median last dose C_max_ was significantly lower for EM (2.60 ng/mL and 2.55 ng/mL) (Figure 10A). However, the overall range of predicted C_max_ was similar to those reported [9]. This difference, however, may be attributed to the anthropometric differences between Southeast Asian/Far East Asian population groups and European (Caucasian) populations [66] in addition to differences in the prevalence of each genotype [23,24,25,26,27,28].

Similar reports for Malaysians subjects are currently lacking. However, in mainland Chinese subjects, clopi-H4 C_max_ for the study duration (i.e., first dose) was reported to be 18.9 ng/mL ± 11.8 ng/mL in EM (*1/*1) and 11.8 ng/mL ± 5.1 ng/mL in PM (*1/*2 or *2/*2) [67], within three-fold of the simulated mean C_max_ for EM (8.62 ng/mL ± 11.4 ng/mL) and PM (5.59 ng/mL ± 3.92 ng/mL) (Figure 10B) whilst also being within a similar range of observed concentrations, when taking into account the standard deviations reported for C_max_ [67].

No significant differences were observed in clopi-H4 plasma concentrations between the Malaysian Chinese and Malay populations when comparing CYP2C19-genotyped groups (Figure 10). This finding is consistent with reports of a similar frequency of CYP2C19 metaboliser groups between the Malay and Malaysian Chinese in a cohort of Malaysian patients taking clopidogrel [29,68,69]. Thus, although the impact of the CYP2C19 polymorphism on clopidogrel pharmacokinetic may lead to treatment failure in PM within the Malay and Malaysian Chinese population, due to the attenuation of clopi-H4 plasma concentration, the magnitude of this impact between these populations is largely minimal, with insignificant differences observed between them (Figure 10).

### 3.4. Step 4: Sensitivity Analysis for CYP2C19 Hepatic Abundances

Despite CYP2C19 polymorphisms having been previously characterised for Malaysians, the hepatic abundance of CYP2C19, and how it varies from Caucasian subjects, is currently lacking. Within the context of pharmacokinetic modelling, this is an important quantitative metric, allowing both the prediction of in vivo clearance (from in vitro hepatocyte/microsomal incubations) and, when combined with appropriate phenotype/genotype data, the ability to model the impact of polymorphisms on resultant drug pharmacokinetics. However, for the Chinese population group, hepatic CYP2C19 abundance has been quantified along with phenotype-specific abundances (8, 0, 6 and 10 pmol/mg protein for EM, PM, IM and UM, respectively), and these have been incorporated into the Simcyp population database and characterised/validated by Simcyp and other researchers [70,71].

However, comparisons to other Asian population groups (e.g., Japanese) show variations in the hepatic abundance for EM phenotypes (14 pmol/mg for Caucasians; 9 pmol/mg for Chinese; 1 pmol/mg for Japanese) [71]. Given this variation, it was prudent to simulate the impact of variation in CYP2C19 EM and PM phenotype abundance, and this was accomplished through applying a 30% increase and 30% decrease of mean abundance values for all CYP2C19-phenotyped groups in the Malay (Figure 11) and Chinese population (Figure 12).

With a 30% increase in mean abundances, there was a slight increase in patient’s response towards clopidogrel treatment based on the clopi-H4 minimum limit of 0.81 ng/mL, ranging from 10% to 20% of the CYP2C19-phenotyped group in both populations. Similarly, with the 30% decrease of mean abundances, a slight decrease of patient’s response can be observed varying between 10% and 30% of the CYP2C19-phenotyped group in both populations. Clearly, these observations are not novel, given that clopi-H4 plasma concentrations are related to the functional status of CYP2C19 within Asian populations [72]. However, of note was the fact that there was also no significant difference in the clopi-H4 C_max_ between the Malay and Malaysian Chinese population groups in relation to the CYP2C19-phenotyped group with the ±30% mean abundance values (Figure 11 and Figure 12), further confirming our earlier findings.

### 3.5. Step 5: Dose Optimisation in CYP2C19 Poor Metabolisers

In the final step, given the high percentage of subjects with a clopi-H4 C_max_ below the minimum therapeutic concentration under standard dosing procedures (300 mg loading dose followed by 75 mg for four days), we assessed the impact of a high-dose regimen on clopi-H4 C_max_. Under these revised dosing conditions, the percentage of subjects with a clopi-H4 C_max_ below the minimum therapeutic concentration decreased to 12% (Malay) and 14% (Malaysian Chinese) (Figure 13). A number of previous clinical studies have considered the high dose versus standard dose clopidogrel treatment regimens, particularly for CYP2C19 PM, and identified no significant clinical concerns, with improved inhibition of platelet aggregation and clinical outcomes [73,74,75,76], and our simulations further agreed with these published findings. Thus, a 600 mg loading dose followed by a 150 mg maintenance dose may be appropriate for confirmed CYP2C19 PM Malay and Malaysian Chinese patients, particularly where platelet response is poor.

### 3.6. Study Limitations and Future Directions for Clopidogrel Use in Malaysia

It is important to address several limitations of the present study. Firstly, although we were able to develop robust pharmacokinetic models, the limited availability of hepatic CYP2C19 abundance data and phenotype/genotype specific abundance data in Malay or Malaysian Chinese was a primary limitation. We utilised existing data from the Chinese population group, which had been previously validated by Simcyp, as a surrogate for both Malay and Malaysian Chinese. It could be possible that inter-ethnic differences exist between Malay and Malaysian Chinese, which may alter the resultant simulations, although no significant differences in clopi-H4 C_max_ were noted in Step 4 (Section 2.4) of our modelling approach.

The focus of this study has been on LOF alleles (e.g., the PM phenotype). However, other SNPs have been reported to contribute towards the overall wide inter-individual variability associated with clopidogrel anti-platelet activity, such as the GOF variant CYP2C19*17 (rs12248560) that defines the ultra-rapid metaboliser phenotype. Whilst the importance of the LOF alleles are well characterised, the GOF variants are less well characterised and their impact of antiplatelet activity is contradictory, as highlighted by a number of meta-analysis studies [77,78,79]. Further modelling studies should investigate the relevance of the UM phenotype on clopi-H4 levels to identify whether the GOF alleles are important for overall clinical efficacy and clopi-H4 plasma concentrations.

Furthermore, there was a lack of published and robust genotyped pharmacokinetic data in Malaysian subjects, primarily plasma concentration–time profiles, which may have aided in model validation of the clopidogrel predictions. However, this has been completed by a prior group in Caucasian subjects [14]. Despite this, further investment in research and development infrastructure is required to ensure that pharmacokinetic modelling approaches are better integrated into clinical research to optimise study design and better utilise the clinical data obtained to provide evidence-based optimised therapy [80].

## 4. Materials and Methods

Population-based PBPK modelling was conducted using the virtual clinical trials simulator Simcyp (Simcyp Ltd., a Certara company, Sheffield, UK, Version 16). Simulations were performed for an exclusive CYP2C19 extensive metaboliser (EM) (CYP2C19*1/*1), poor metaboliser (PM) (CYP2C19*2/*2), intermediate metaboliser (IM) (CYP2C19*1/*2) and ultrarapid metaboliser (UM) (CYP2C19*1/*17) population groups. For all simulations, dosing occurred under fasted-conditions unless otherwise indicated. A detailed list of haplotypes associated with GOF or LOF alleles are detailed within the PharmGKB database (Accession number: PA124) [19].

### 4.1. Model Development

**Figure 14:**
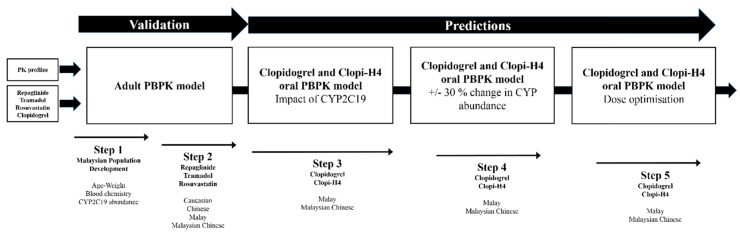
Model development strategy. The five-stage workflow approach implemented to study pharmacokinetics within Malaysian population groups.

A five-stage stepwise approach was implemented for model development, validation and model refinement (Figure 14), which is fully described below.

Phenotype frequencies for CYP2C19 were incorporated for Caucasian (EM: 97.6%; PM: 2.4%) and Chinese (EM: 87%; PM: 13%) populations using the default frequencies recorded within Simcyp. Literature reported frequencies were utilised for Malay (EM: 51%; PM: 7%; IM: 38%; UM: 4%) [81].

#### 4.1.1. Step 1: Malaysian Population Development

To develop a Malaysian population group for use in pharmacokinetic modelling, the National Cardiovascular Database (NCVD) Registry [6] was analysed for relevant population-level anthropometric data relevant to each ethnic group. The NCVD register is a Malaysian nationwide registry consisting of 33,043 anonymized and voluntary patient records for patients undergoing acute coronary syndrome and percutaneous coronary intervention, spanning the years 2006–2015. The NCVD is supported by the Ministry of Health Malaysia and co-sponsored by National Heart Association of Malaysia, with the aim to gather information about cardiovascular diseases in Malaysia. Within this database, relevant physiological parameters were limited to only (i) gender; (ii) age; (iii) weight and (iv) ethnicity.

To develop the Malaysian population group the two largest ethnic groups, Malays and Malaysian Chinese were considered, as they constitute 50.1% and 22.6% of the total Malaysian population, respectively [1]. Appropriate anthropometric age–body weight distributions were generated and used to establish mathematical (polynomial regression) relationships to predict body weight from age, using TableCurve2D (version, Systat Software, San Jose, CA, USA). The resultant polynomial regression equations were then applied within the population ‘Demographics’ section of Simcyp to create user-defined age–weight relationships for each population. Furthermore, blood chemistry was revised to match report haematocrit and plasma protein concentrations within the Malay and Malaysian Chinese population groups, as reported in the literature (Table 3).

In the absence of literature reported CYP2C19 hepatic abundance in the Malay and Malaysian Chinese subjects, the EM, PM, IM and UM phenotypes were allocated a hepatic abundance of 8, 0, 6 and 10 pmol/mg protein, respectively, based upon adaptations detailed within a validated Chinese population group developed by Simcyp, and which is available from the population library repository of Simcyp software. These abundances were assumed to be the same for both Malay and Malaysian Chinese population groups.

##### Compound Selection and Clinical Studies

A literature search for published clinical studies reporting plasma concentration–time profiles for ethnicity-specific Malaysian patients (Malay and Chinese) was able to identify repaglinide, tramadol, rosuvastatin and clopidogrel as therapeutic drugs where pharmacokinetic clinical data was available for population groups of interest. These studies were used for model development and validation. The three compounds of interest had previously been developed and validated within Simcyp and are available within the Simcyp library compound database, with repaglinide developed and pre-validated by Simcyp [82], tramadol developed and validated by T’jollyn et al. [47] and clopidogrel and its’ primary metabolite (2-oxo-clopidogrel) and secondary metabolite (clopi-H4) previously developed and validated by Djebli et al. [14].

#### 4.1.2. Step 2: Adult Simulations: Validation with Repaglinide, Tramadol and Rosuvastatin

To confirm the validity of the modelling approaches and the appropriateness of the customised Malaysian population groups, validation of the population group was conducted using repaglinide, tramadol and rosuvastatin from six published clinical studies: (i) a single 2 mg oral dose of repaglinide to healthy adult volunteers [42]; (ii) a single 2 mg oral dose of repaglinide on day 1 and subsequently 2 mg multiple doses orally on day 2 to day 9 in healthy young adults [43]; (iii) a single 2 mg oral dose of repaglinide dosed to healthy native Han Chinese adult volunteers [44]; (iv) a single 4 mg oral dose of repaglinide dosed to healthy adult Malay volunteers [45]; (v) a single dose of 100 mg tramadol given intravenously to adult mixed Malaysian volunteers [46] and (vi) a single oral dose of 40 mg rosuvastatin given Caucasian, Han Chinese and Malays [48] with appropriate hepatic uptake clearances incorporated as reported by Bae et al. [83]. All simulations replicated the study design reported by the clinical studies cited above.

#### 4.1.3. Step 3: Prediction of the Impact of CYP2C19 Polymorphisms on Clopidogrel Pharmacokinetics in Malaysians

This step simulated the potential impact of SNPs CYP2C19 on the resultant clopi-H4 target plasma concentration range for patients known to result in a clinical response, namely 0.81 to 13.45 ng/mL [49], within the Malay and Malaysian Chinese population groups. For all simulation, clopi-H4 concentration of below 0.81 ng/mL was used as a cut-off value to depict patients who were unresponsive to clopidogrel treatment. Simulations were stratified across EM, PM, IM and UM phenotypes, and performed using a validated clopidogrel compound (18) using a trial design of 63 adult subjects who were administered a 300-mg loading dose (LD) and a 75-mg/day maintenance dose (MD) for four days.

#### 4.1.4. Step 4: Sensitivity Analysis for CYP2C19 Hepatic Abundances

To address the absence of literature reported CYP2C19 hepatic abundances in the Malaysian population groups, two further scenarios were simulated whereby the mean abundances for the Malay and Malaysian Chinese population were set at 30% greater/less than that used as default (see Step 1). This allowed for the analysis of the sensitivity of model predictions to changes in abundance to be simulated through assessing the resulting impact on the number of subjects attaining clopi-H4 target concentrations.

#### 4.1.5. Step 5: Dose Optimisation in CYP2C19 Poor Metabolisers

This step attempted to predict the potential impact of dosage optimisation in the CYP2C19 poor metabolisers’ population, with an aim to recapitulate subjects into the clopi-H4 therapeutic window range. Simulations were run using the Malay and Malaysian Chinese population groups. Furthermore, based on the study by Simon et al. [32], the dosing regimen for clopidogrel was increased to a ‘high-dose’ scenario with a 600 mg loading dose followed by a four-day regimen of 150 mg daily.

### 4.2. Data Analysis

Clinical plasma concentration–time data points from studies identified in Step 1 (Section 4.1.1) were extracted using the WebPlotDigitizer v.3.10 (http://arohatgi.info/WebPlotDigitizer/). All simulations of plasma concentration–time profiles were presented in 5th to 95th percentiles and either in mean or median unless otherwise specified. For all adult simulations, age ranges and subject gender ratios were matched, where possible, to reported clinical studies. Where this information was not cited in clinical studies, a default age range of 40 to 65 years and gender ratio of 50% was selected. For simulations employing genotypes stratification, unless otherwise stated, a 100-subject simulation was run in a 10 × 10 trial (10 subjects per trial with 10 trials) per genotype to ensure that reasonable inter-/intra individual variability is captured within the model simulations.

### 4.3. Predictive Performance

In all simulations, a prediction to within two-fold of the observed data was generally accepted as part of the ‘optimal’ predictive performances range despite there being no uniform standard of acceptance to determine this criterion [84,85,86]. This acceptance criterion was used in our C_max_ and AUC comparisons with the published clinical data reported. However, this was not used as the sole determinate for model performance (see Section 4.4).

For the clopidogrel simulations, the target clopi-H4 plasma concentration was set at the lowest value of 0.81 ng/mL from the range of 0.81–13.45 ng/mL obtained from literature [49] and used to determine the impact of SNPs CYP2C19 on Malay and Malaysian Chinese population pharmacokinetics.

### 4.4. Visual Predictive Checks

To further validate model predictions where comparison were made to existing clinical studies, a visual predictive checking (VPC) strategy was adopted. This approach was described at the 2012 FDA Pediatric Advisory Committee (US Food and Drug Administration, 2012) [87]. In this approach, to graphically validate the predictability of the model, the 5th and 95th percentiles (along with mean or median) of predicted concentration–time profiles (generated from Simcyp) were graphically displayed along with the observed data for any validation data sets to ensure predicted data points largely overlapped with those from the observed data sets, which should contain (where possible) some measure of spread of observed plasma concentration data (e.g., a standard deviation for each mean concentration point).

## 5. Conclusions

Cardiovascular disease (CVD) is a leading cause of mortality and is increasingly prevalent in Malaysia, which places the Malaysian healthcare system at ever increasing risks and cost-burdens for treatment of patients. Given the unique ethnic diversity of the Malaysian population group, evidence-based approaches should account for the individual characteristics of patients rather than focusing on an average patient from a carefully selected patient population. Pharmacokinetic modelling can provide this approach through carefully developed and validated population models which can be applied to study a drug’s pharmacokinetics in different geographical regions. This approach was applied to clopidogrel and illustrated the impact of a PM phenotype on reducing clopi-H4 C_max_, which could be partially recovered using a high-dose strategy (600 mg loading dose followed by 150 mg maintenance dose), which resulted in an approximate 50% increase in subjects attaining the minimum clopi-H4 plasma concentration for a therapeutic effect. Furthermore, we illustrated limited variation clopi-H4 pharmacokinetics between the two key ethnic groups, Malays and Malaysian Chinese, suggesting that inter-ethnic differences within Malaysia may not impact upon clopidogrel therapy. It should be noted, however, that the current lack of existing clopidogrel pharmacokinetics data in multi-ethnic population groups is lacking within Malaysian populations, and this data would be valuable to support the work presented within this manuscript and to also aid in validating the outcomes presented.

This study has illustrated the benefit of the application of pharmacokinetic modelling, which incorporates pharmacogenetics information, to mixed ethnic population groups. The current lack of an integrated approach within Malaysia, in addition to the sparse routine application of pharmacokinetic modelling to clinical data, should be addressed to support better clinical drug decision-making.

## Figures and Tables

**Table 1:** Summary demographic data from the NCVD database.

Ethnicity		Age (years)	Height (m)	Weight (kg)	BMI (kg/m^2^)
Malay	Mean	57.75	1.63	69.79	26.19
	Median	57.6	1.63	69.5	25.78
	N	23114	10250	12193	10170
	SD	11.62	0.08	14.24	4.35
Malaysian Chinese	Mean	62.89	1.63	67.17	25.11
	Median	63.1	1.63	66	24.79
	N	9929	4111	5259	4086
	SD	12.04	0.08	13.18	3.84
Malaysian Indian	Mean	57.7	1.63	69.61	25.95
	Median	57.3	1.64	69	25.52
	N	9167	4257	4809	4229
	SD	11.91	0.09	13.91	4.25

N: total number of recorded metrics; SD: standard deviation.

**Table 2:** Summary of predicted and observed pharmacokinetic parameters of repaglinide, tramadol and rosuvastatin.

Compound	Validation	CL (L/h)		C_max_ (ng/mL)		t_max_ (h)		AUC (ng/mL·h)	
		Observed	Predicted	Observed	Predicted	Observed	Predicted	Observed	Predicted
Repaglinide	Hatorp et al., (2002) (Healthy volunteers)	-	12.17 (10.03–14.77)	26.0 µg/L	32.9 (16.4–47.0) µg/L	0.83 (0.31–1.35)	0.54 (0.18 -0.91)	152.4 (62.8–242.0) µg/L·h	70.89 (15.51–73.82) µg/L·h
	Hatorp et al., (1999) (Single dose) (Healthy volunteers)	-	13.9 (10.33–18.70)	47.9 (15.9–79.9)	26.2 (19.61–35.0)	0.8 (0.2–1.4)	0.47 (0.31–0.60)	69.0 (61.2–76.8)	54.87 (21.57–106.1)
	Hatorp et al., (1999) (Multiple doses) (Healthy volunteers)	-	15.32 (12.24–20.57)	58.5 (8.1–108.9)	26.39 (11.53–46.85)	0.6 (0.5–0.7)	0.48 (0.32–0.61)	98.1 (84.54–111.66)	56.8 (22.59–109.1)
	Zhai et al., (2013) (Chinese) ^1^	-	56.66 (21.10–146.00) (Malaysian Chinese)	20.0 (14.9–25.1)	18.5 (8.79–28.0) (Malaysian Chinese)	1.2 (0.5–1.9)	0.93 (0.82–1.08) (Malaysian Chinese)	46.3 (31.2–61.4)	39.51 (16.21–74.56) (Malaysian Chinese)
	Ruzilawati et al., (2010) (Malay)	11.82 (7.86–15.78)	10.41 (5.29–15.53)	83.56 (55.63–111.49)	67.5 (34.4–118)	0.62 (0.24–1.00)	0.58 (0.20–0.96)	340.66 (226.14–455.18)	151 (33.61–196)
Tramadol	Gan et al., (2002) ^2^ (Malaysian)	19.3 (13.1–25.5)	19.24 (13.31–33.11) (Malay)	-	-	-	-	5078.4 (3117.3–7039.5)	4389 (2915–6128) (Malay)
			18.36 (11.73–30.19) (Malaysian Chinese)	-	-	-	-		4716 (3201–6658) (Malaysian Chinese)
Rosuvastatin	Lee et al., (2005) (Malay, Chinese, Healthy volunteers Caucasians)	-	21.87 (19.49–24.53) (Malay)	50.0 (42.2–59.3) (Malay)	49.91 (42.68–58.36) (Malay)	3.00 (0.50–5.00) (Malay)	3.01 (2.51–3.61) (Malay)	413 (354–482) (Malay)	552.84 (473.51–645.46) (Malay)
			21.28 (19.17–23.63) (Chinese)	59.1 (49.8–70.1) (Chinese)	53.83 (42.01–57.35) (Chinese)	3.00 (0.50–5.00) (Chinese)	2.90 (2.43–3.45) (Chinese)	500 (428–583) (Chinese)	553.18 (475.13–644.05) (Chinese)
			23.99 (22.40–25.70) (Healthy volunteers)	25.0 (21.1–29.6)	13.49 (10.55–14.28)	5.00 (1.00–8.00)	2.99 (2.15–3.16)	216 (186–252)	116.15 (98.70–136.68)

Data presented as mean (range). ^1^ Validation study was reported within a Chinese population group. Simulations were performed within a Malaysian Chinese group for comparison; ^2^ Validation study was reported within a Malaysian population group without demarking ethnicities. Simulations were performed in Malay and Malaysian Chinese for comparison. CL: Oral clearance; C_max_: maximum plasma concentration; t_max_: time to maximum plasma concentration; AUC: area under the plasma concentration–time curve.

**Table 3:** Malay and Malaysian Chinese blood biochemistry.

Biochemistry	Malay	Malaysian Chinese
Haematocrit (%)	M: 43 ^b^ F: 38 ^c^	M: 45.3 F: 40.5
AAG (g/L)	M: 0.65 ^a^ F: 0.64 ^a^	M: 0.65 F: 0.64
HSA (g/L)	M: 47.3 ^b^ F: 46.3 ^b^	M: 50.34 F: 49.38

AAG: α1-acidic glycoprotein; HSA: human serum albumin M: male; F: female; ^a^ Simcyp default values; ^b^ Hamzah et al. [64]; ^c^ Khor et al. [65].
